# Coumarin derivative-functionalized nanoporous silica as an on–off fluorescent sensor for detecting Fe^3+^ and Hg^2+^ ions: a circuit logic gate

**DOI:** 10.1186/s11671-024-04013-9

**Published:** 2024-04-22

**Authors:** Zahra Mousavi, Jahan B. Ghasemi, Ghodsi Mohammadi Ziarani, Shahnaz Rahimi, Alireza Badiei

**Affiliations:** 1 School of Chemistry, College of Science, University of Tehran, P.O. Box: 14155-6455, Tehran, Iran; 2 Department of Organic Chemistry, Faculty of Chemistry, Alzahra University, P.O. Box: 1993893973, Tehran, Iran

**Keywords:** Nanoporous silica, Fluorescent sensor, Fe^3+^ ions, Hg^2+^ ions, Logic gate, SBA-15

## Abstract

A highly efficient fluorescent sensor (S-DAC) was easily created by functionalizing the SBA-15 surface with N-(2-Aminoethyl)-3-Aminopropyltrimethoxysilane followed by the covalent attachment of 7-diethylamino 3-acetyl coumarin (DAC). This chemosensor (S-DAC) demonstrates selective and sensitive recognition of Fe^3+^ and Hg^2+^ in water-based solutions, with detection limits of 0.28 × 10^–9^ M and 0.2 × 10^–9^ M for Hg^2+^ and Fe^3+^, respectively. The sensor’s fluorescence characteristics were examined in the presence of various metal ions, revealing a decrease in fluorescence intensity upon adding Fe^3+^ or Hg^2+^ ions at an emission wavelength of 400 nm. This sensor was also able to detect ferric and mercury ions in spinach and tuna fish. The quenching mechanism of S-DAC was investigated using UV–vis spectroscopy, which confirmed a static-type mechanism for fluorescence quenching. Moreovre, the decrease in fluorescence intensity caused by mercury and ferric ions can be reversed using trisodium citrate dihydrate and EDTA as masking agents, respectively. As a result, a circuit logic gate was designed using Hg^2+^, Fe^3+^, trisodium citrate dihydrate, and EDTA as inputs and the quenched fluorescence emission as the output.

**Graphic abstract:**

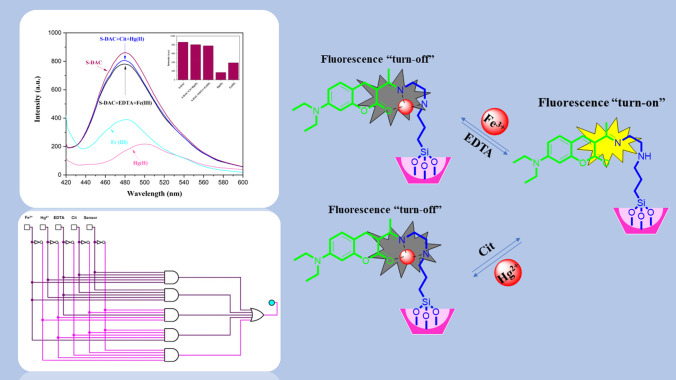

**Supplementary Information:**

The online version contains supplementary material available at 10.1186/s11671-024-04013-9.

## Introduction

Today, there is evidence that living organisms' water environment and food cycle are contaminated with non-biodegradable hazardous heavy metal ions. Therefore, due to their biological effects on living systems, the identification of these pollutants has received wide attention from researchers [1–3]. Although mercury metal exists naturally in the environment, such as air, water, soil, and organisms are exposed to harmless low levels, critical doses are associated with adverse physical and neurotoxic side effects in living organisms [4–8]. Among hazardous compounds, mercury ranks as the sixth most toxic element [9]. While all types of Mercury, including elemental (Hg^0^) and ionic (Hg^+^, Hg^2+^), are toxic and bioaccumulated, methyl mercury tends to build up more significantly than other forms. Bacteria can transform elemental (Hg^0^) and ionic Mercury (Hg^+^, Hg^2+^) into methyl mercury, which then enters the food chain of water-dwelling organisms and bioaccumulates over time [10–12].

Monitoring and researching pollution in water-dwelling organisms like fish and sea mammals, along with the subsequent harm, results in worldwide expenses amounting to billions [13].

Accumulation of both organic and inorganic forms Mercury species in the human body can lead to a variety of diseases related to the brain and nervous system, such as irritability, nervousness, changes in personality, sensory issues like vision problems and deafness, and cognitive disorders. It can also cause Minamata disease, and in severe cases, it could be fatal [6–8, 14–17].

Fe^3+^, as one of the most abundant heavy metals in the Earth’s crust, plays an essential role in various biochemical processes at the cellular level in most organisms [18–20]. The role of Fe^3+^ in these biochemical processes includes DNA and RNA replication, facilitating the oxygen transfer from lungs to tissues via heme, regulation of cellular electron transactions, and different bio-syntheses such as enzymatic reactions, and cellular metabolism [21–23]. However, the level of Fe^3+^ in the organism is crucial, with lower and higher levels linked to different diseases and even life-threatening conditions. For instance, insufficient Fe^3+^ is associated with anemia, diabetes, cancer, hemochromatosis, dysfunctions in the liver and kidney, and neurogenerative diseases such as Parkinson's and Alzheimer's [20, 24–30]. On the other hand, an elevated concentration of Fe^3+^ is linked to the creation of free radicals, which can harm tissues and escalate the oxidation of cellular components like lipids and proteins [29–31]. The scientific community has extensively researched the monitoring and detection of Fe^3+^ using various sensors. A range of methods have been employed to detect heavy metals [32–36]. On the other hand, the fluorescent sensors have been considerably developed due to their low costs, easy operation, high sensitivity, selectivity, and rapid response [37–39]. Fluorescent sensors based on organic molecules are among the most common fluorescent sensors [40].

Although most of these sensors need organic solvents for optimal performance and are not soluble in H_2_O, their use in entirely aqueous media is limited. Therefore, there is a pressing need to design and produce a new class of fluorescent sensors to overcome these limitations [41–44].

Recently, organic–inorganic hybrid sensors have been developed based on fixing fluorophore groups onto the surface of inorganic nanomaterial substrates. Among, Nanoporous silica materials, SBA-15 is particularly notable due to its unique features such as hydrothermal stability, uniform pores structure, thick walls, high specific surface area, and biocompatibility. Additionally, the optically transparency and non-fluorescence nature of SBA-15 make it an suitable candidate for grafting with fluorophores to create fluorescent sensors [45–47]. Herein,

a highly efficient fluorescent sensor (S-DAC) is easily created by functionalizing the SBA-15 surface with N-(2-Aminoethyl)-3-Aminopropyltrimethoxysilane followed by the covalent attachment of 7-diethylamino 3-acetyl coumarin (DAC).

## Experimental part

Information about materials and instruments is available in Supplementary data.

The SBA-15 [48], SBA-Pr-NH-Et-NH_2_ [49] and 7-diethylamino 3-acetyl coumarin (DAC) [50] were synthesized as explained in the mentioned references.

## Surface modification of SBA-Pr-NH-Et-NH_2_ with 7-diethylamino 3-acetyl coumarin (S-DAC)

Firstly, the 7-diethylamino-3-acetyl coumarin compound (5 mmol) was dissolved in EtOH (25 ml), added to the suspension of SBA-Pr-NH-Et-NH_2_ (1 g) in EtOH (25 ml), and refluxed for 24 h. After the filteration of reaction mixture, the filterate was washed with EtOH and then EtOAc to obtain the final product of S-DAC, which was charechterized by different methods.

### Fluorescence measurements

For all fluorescence experiments, 0.005 g of S-DAC in 100 ml of deionized water was dispersed using an ultrasonic bath. Suitable nitrate salts were dissolved in distilled water to prepare stock solutions of metal ions (1 × 10^–2^ mol L^−1^). In addition, sodium and potassium salts were employed to prepare the anionic stock solutions (1 × 10^–2^ mol L^−1^). 2 mL of the dispersed solution and 50 µL of various metal ions (10^−2^ M) were used to record the fluorescence spectrum. For the competition tests, besides the target ion, other ions as interfering agents with a ratio of 1:5 were added to the 2 mL of the dispersed solution. Furthermore, for the titration measurements, different amounts of the target ion (0–200 µL, 1 × 10^–6^ mol L^−1^) were added to the 2 mL of S-DAC dispersion. The photoluminescence measurements were recorded with the emission wavelength at about 480 nm, while the excitation wavelength was tuned at 400 nm.

## Results and discussion

This study presents a fluorescent switchable sensor based on a coumarin derivative-functionalized SBA-15 for selective recognition of Hg^2+^ and Fe^3+^ in the aqueous environment and real sample. The design of this type of mesopore-based sensor has shown several results. Firstly, it increases the surface area, allowing more fluorophores to be loaded. Secondly, it reduces the detection limit or increases its sensitivity. The chemosensor of S-DAC comprises 7-diethylamino 3-acetyl coumarin (DAC) units as a fluorophore and N-(2-Aminoethyl)-3-Aminopropyltrimethoxysilane as a linker, on a mesoporous substrate. It was evaluated its photoluminescence performance in the presence of various metal ions. The obtained results revealed that the fluorescence intensity of S-DAC was remarkably quenched by Hg^2+^ and Fe^3+^ ions compared to other cations. Further investigation showed that trisodium citrate dihydrate and EDTA could reverse the emission intensity of mercury and ferric ions, respectively, and a logic gate circuit was provided. Finally, this potential chemosensor can be applied in real samples such as spinach and tuna fish.

### Functionalization of SBA-Pr-NH-Et-NH_2_ with coumarin derivatives

**Scheme 1:**
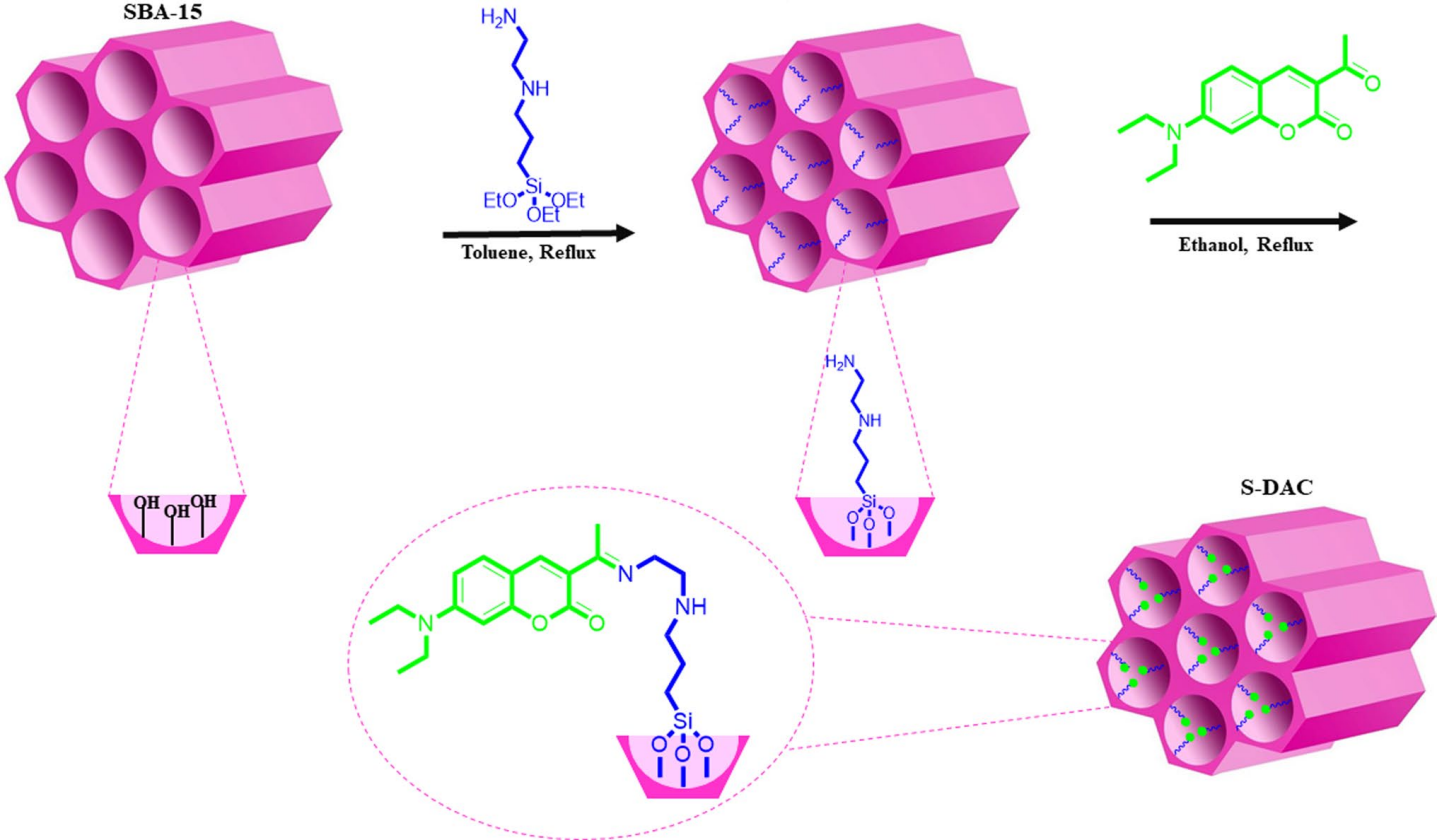
The illustration of the synthetic S-DAC process

Coumarin derivative-functionalized nanoporous silica (S-DAC) was easily created by functionalizing the SBA-15 surface with N-(2-Aminoethyl)-3-Aminopropyltrimethoxysilane, followed by the reaction with 7-diethylamino 3-acetyl coumarin (DAC), which was prepared by the.reaction of 4-diethylamino salicylic aldehyde, acetyl acetoacetate and piperidine in EtOH as depicted in Scheme 1.

### Structural characterization of S-DAC

**Fig. 1:**
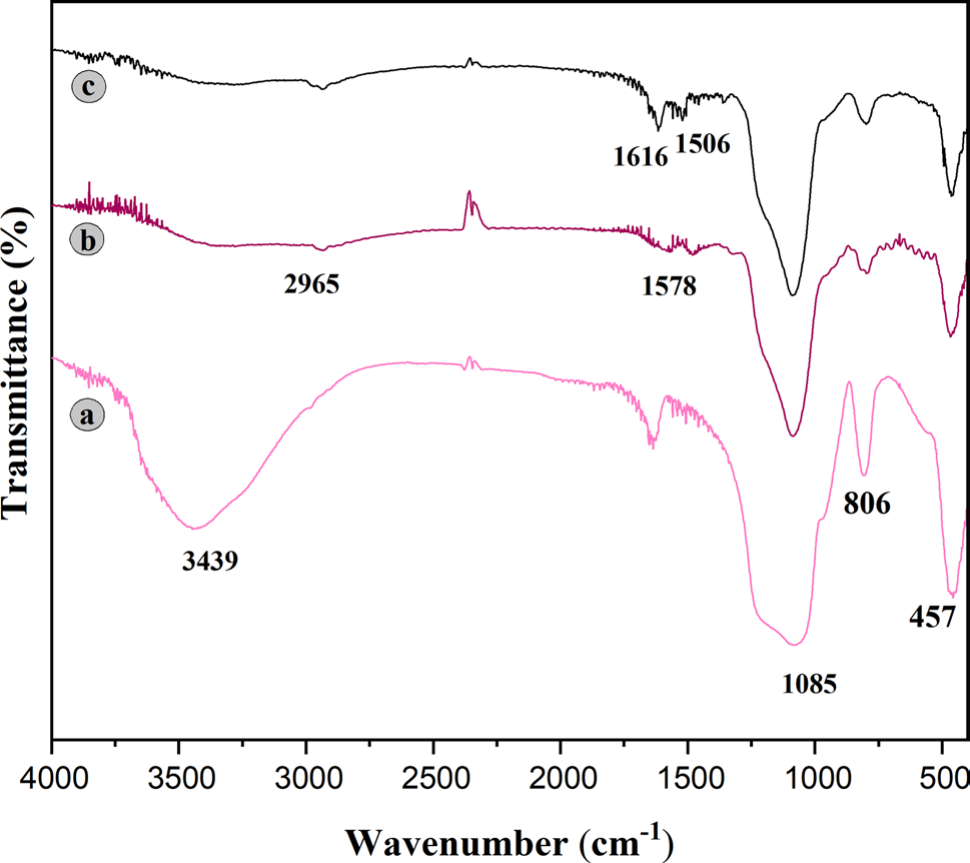
FT-IR spectra of **a** SBA-15, **b** SBA-Pr-NH-Et-NH_2_, **c** S-DAC

FT-IR spectroscopy was utilized to confirm the successful immobilization of functional groups onto the pore walls of SBA-15. The FT-IR spectra of SBA-15, SBA-Pr-NH-Et-NH_2,_ and S-DAC are presented in Fig. 1. For all samples, the bands located at around 457 cm^−1^, 1806 cm^−1^, and 1085 cm^−1^ are related to bending vibrations and symmetric and asymmetric stretching vibrations of siloxane (Si–O–Si) groups, respectively [49]. The band at around 973 cm^−1^ is attributed to the stretching vibrations of the silanol groups (Si–OH) [51]. The broad peak around 3439 cm^−1^ is assigned to the stretching vibrations of the –OH groups belonging to H_2_O and silanol groups [49, 51]. The band at around 1635 cm^−1^ is related to the physically absorbed H_2_O molecules [52]. The intensity of the peaks associated with silanol groups (973 cm^−1^ and 3439 cm^−1^) decreases after grafting with the linker. The appearance of peaks at around 2850–2965 cm^−1^ is related to the aliphatic chain’s stretching vibrations of methylene groups [53]. A new band at about 1578 cm^−1^ is observed, which is probably related to the bending vibration of NH_2_ [51]. In addition, in the spectrum of S-DAC, the strong peak at around 1616 cm^−1^ can be assigned to stretching vibrations of the carbonyl group. Also, two bands at about 1361 cm^−1^ and 1506 cm^−1^ are attributed to the ring C=C and C=N stretching vibrations, respectively [54]. Therefore, the FT-IR results suggest the successful attachment of the fluorophore onto the SBA-15 surface.

**Fig. 2:**
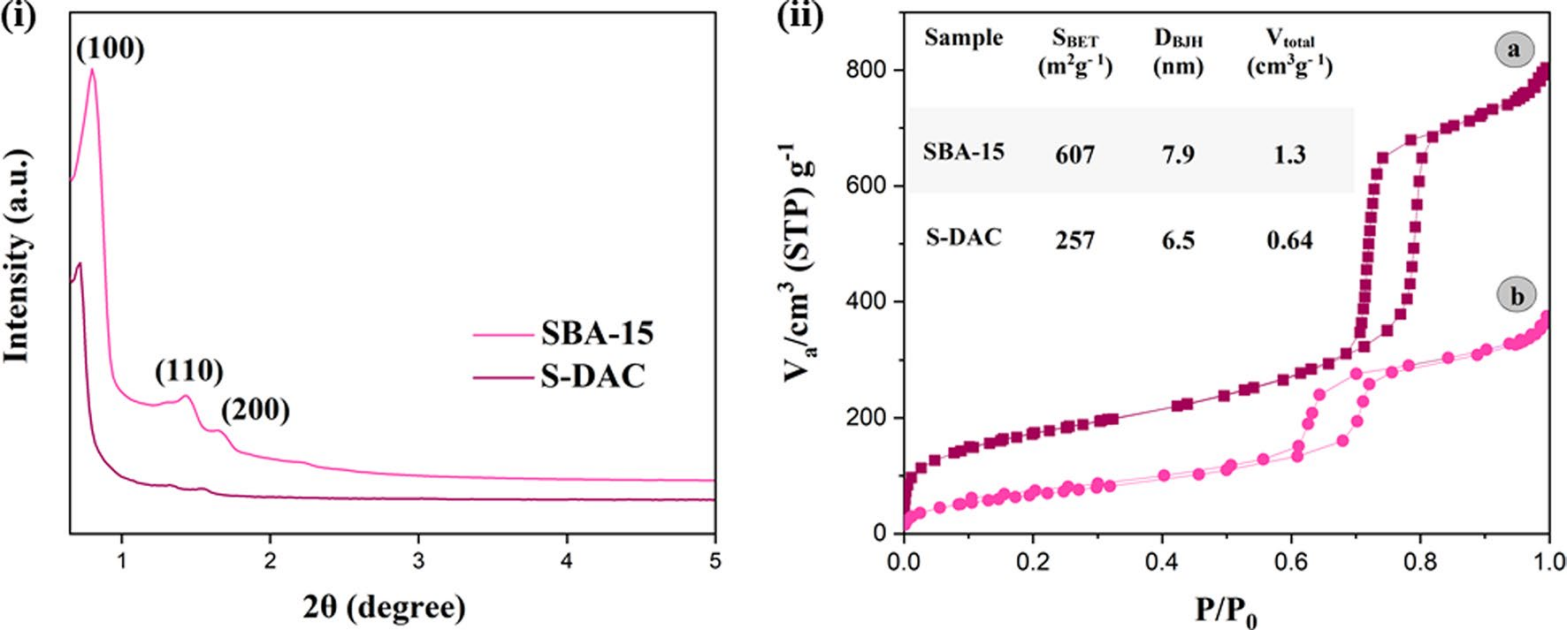
(**i**) Low-angle XRD patterns of SBA-15 and SBA-S-DAC and (**ii**) N_2_ adsorption–desorption isotherms for SBA-15 and S-DAC (inset: textural properties of SBA-15 and S-DAC)

The Low-angle XRD patterns of SBA-Pr-NH-Et-NH_2_ and S-DAC were shown in Fig. 2i. Both samples exhibit three characteristic diffraction patterns related to nanoporous compounds. The intense diffraction near 2θ = 1° diffracted from the (100) plane, accompanied by two weaker peaks near 2θ = 2° diffracted from the (110) and (200) planes, confirms the long-range periodic order and two-dimensional hexagonal mesostructure of SBA-15 with P6mm space group [55]. The similarity of the diffraction patterns in both samples suggests that the original structure of SBA-15 is preserved after the surface modification. The decrease in X-ray diffraction intensity after functionalization is due to the attachment of organic moieties onto the pore walls of SBA-15, which causes a decline in relatively lower long-range order and crystallinity [56]. These results demonstrate the successful surface modification and preservation of the mesostructure after functionalization.

The N_2_ adsorption–desorption isotherms of SBA-15 and S-DAC are presented in Fig. 2ii. The type IV standard IUPAC isotherm with an H1-type hysteresis loop, corresponding to the ordered structure of mesoporous materials, is demonstrated for SBA-15 and S-DAC [57]. The observation of similar characteristics in the isotherm of SBA-15 and S-DAC, but with a lower height of the hysteresis loop and a lower level of gas adsorption in S-DAC, proves that the SBA-15 structure is preserved and the pore walls of S-DAC were not filled or closed after the modification steps. As observed in the inset table of Fig. 2ii, grafting organic moieties onto the walls of SBA-15 channels causes a decrease in the specific surface area (S_BET_), average pore diameter (d_P_), and pore volume (V_P_) of S-DAC.

**Fig. 3:**
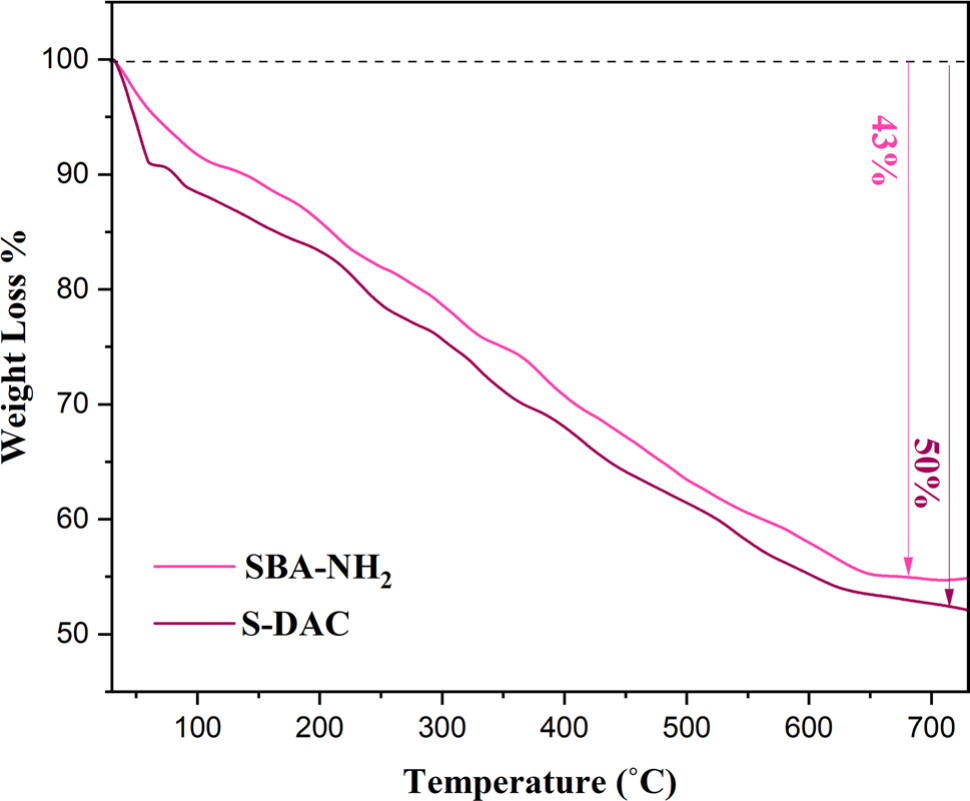
TGA curves of S-DAC and SBA-Pr-NH-Et-NH_2_

The thermogravimetric analysis (TGA) curves of SBA-Pr-NH-Et-NH_2_ and S-DAC were provided in Fig. 3. These curves were used to estimate the approximate amount of the organic moieties grafted to the pore walls of SBA-15. In both cases, the initial mass loss (12%) below 150 °C is attributed to the desorption of the physically adsorbed H_2_O molecules and other volatiles entrapped within the SBA-15 pore channels. Moreover, the subsequent significant mass loss between 150 and 650 °C can be associated with the degradation of grafted organic compounds. The weight loss above 650 °C can be assigned to the dehydroxylation of the Si–OH groups from the SBA-15 framework. The total weight loss for SBA-Pr-NH-Et-NH_2_ and S-DAC was estimated to be about 43% and 50%, respectively. A difference of about 7% between the two curves was attributed to the amount of grafted various organic moieties. As a result, the amount of the attached organic moieties onto the pore channels of SBA-Pr-NH-Et-NH_2_ and S-DAC was estimated at approximately 1.7 and 0.85 mmol g^−1^, respectively.

**Fig. 4:**
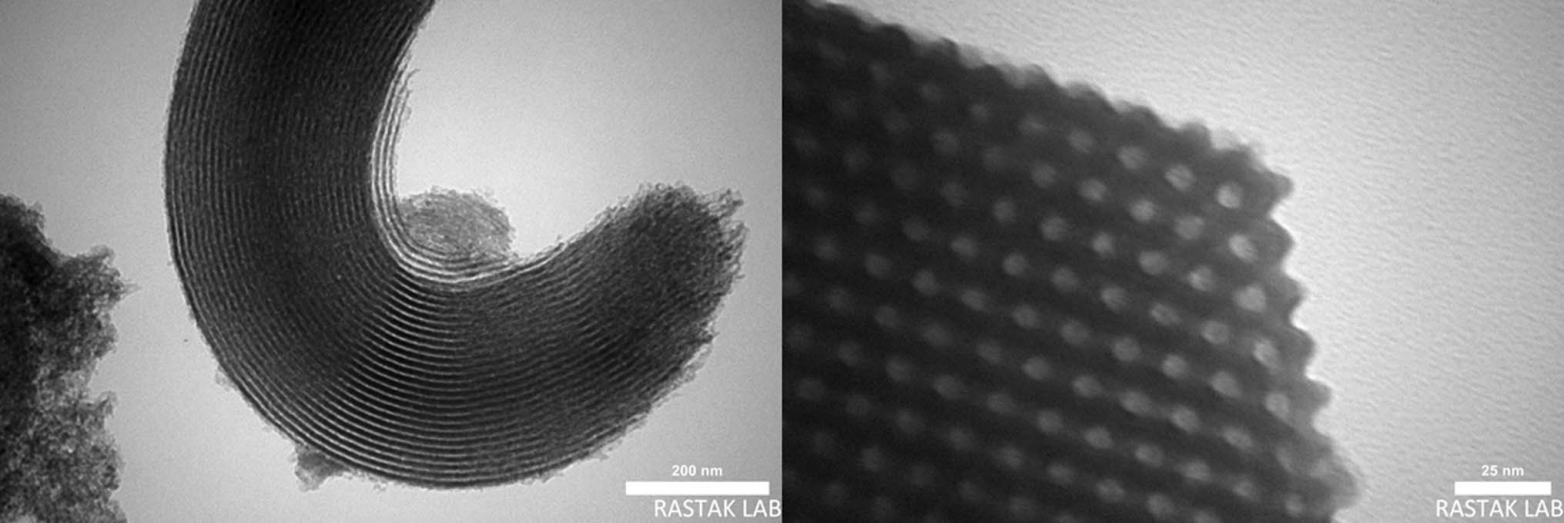
TEM images of S-DAC

The transmission electron microscopy (TEM) images of the S-DAC was demondstrated in Fig. 4, which was exhibited the well-ordered 2D hexagonal rod-shaped mesopore of the S-DAC. This result confirmed that after the grafting of organic molecules, the ordered mesoporous structure of the S-DAC was well maintained.

The Zeta potential of samples in the presence and absence of analytes and particle size distribution of S-DAC can be shown in Table 1.

**Table 1:** Zeta potential of samples in the presence and absence of analytes and particle size distribution of S-DAC

Samples	S-DAC	S-DAC + Fe^3+^	S-DAC + Hg^2+^
Zeta potential (mV)	39.3	50.8	29.4
DLS	693.5 nm		

### Fluorescence studies

#### Fluorescence response of S-DAC to metal ions

**Fig. 5:**
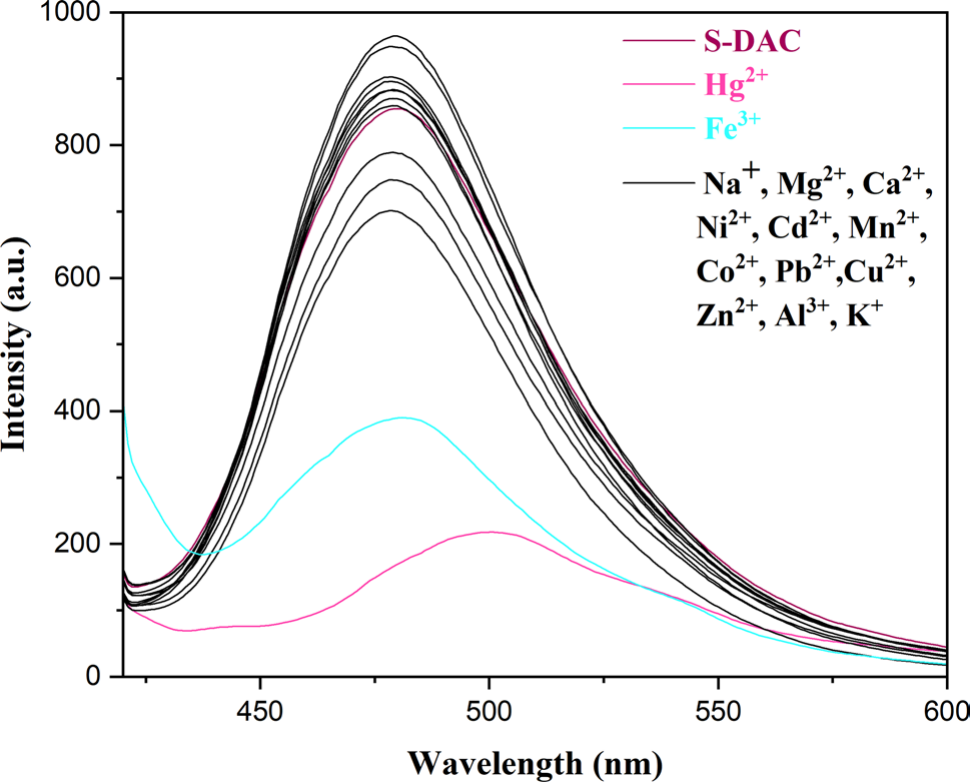
Fluorescence emission of S-DAC (5 × 10^−2^ M) in the presence of different cations (50 μL of [M^n+^]1 × 10^−2^ M) including Hg^2+^, Cu^2+^, Ca^2+^, Zn^2+^, Co^2+^, Cd^2+^, Ni^2+^, Pb^2+^, Fe^3+^, etc. in water, (λ_ex_: 400 nm and λ_em_: 480 nm)

To evaluate the selectivity of the S-DAC fluorescent sensor in water, its fluorescence response was investigated towards a wide variety of metal ions, such as Ca^2+^, Zn^2+^, Co^2+^, Cd^2+^, Ni^2+^, Pb^2+^, Ba^2+^, K^+^, Fe^3+^, Hg^2+^, Cu^2+^, Li^+^, etc. As shown in Fig. 5, the fluorescence spectrum of the free S-DAC (3 ml, 0.05 g L^−1^) was recorded at an excitation wavelength of 400 nm and an emission wavelength of 482 nm. Then, the emission spectrum of the fluorescent sensor was recorded in the presence of various metal ions (50 µL, 10^–2^ M). The fluorescence emission of the S-DAC is significantly quenched upon adding Fe^3+^ and Hg^2+^ ions. In contrast, introducing other metal ions exhibits no notable spectral change or variation of emission intensity. Therefore, the S-DAC fluorescent sensor exhibited excellent selectivity towards Fe^3+^ and Hg^2+^ ions in aqueous solutions.

#### Selectivity study of S-DAC for Fe^3+^ and Hg^2+^ ions

**Fig. 6:**
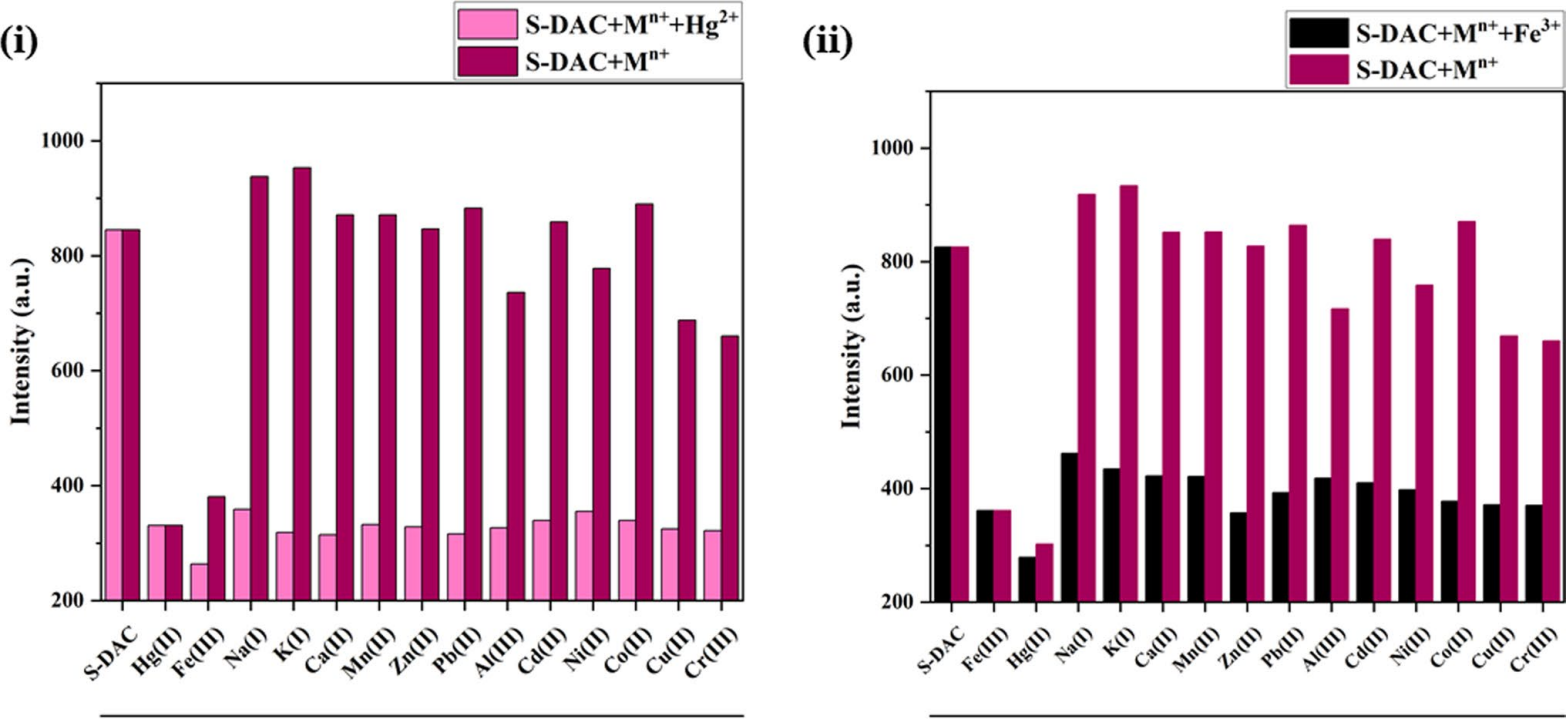
(**i**) Fluorescence emission of S-DAC upon addition of various aqueous metal ions (5 eq.) in the absence and presence of Hg^2+^ (1 eq.) with λ_ex_: 400 nm and λ_em_: 480 nm and (**ii**) Fluorescence emission of S-DAC upon addition of various aqueous metal ions (5 eq.) in the absence and presence of Fe^3+^ (1 eq.) with λ_ex_: 400 nm and λ_em_: 480 nm

In addition, in the presence of other interfering cations, Hg^2+^ and Fe^3+^ ions still bring about similar fluorescence changes. Competition tests were carried out to prove the selectivity of the S-DAC fluorescent sensor towards Hg^2+^ and Fe^3+^ ions. In each experiment, the fluorescence emission intensity was recorded after adding the fluorescent sensor to the mixture of target ions and other interfering ions at a ratio of 1:5. The results show that other cations have no significant effect on the emission intensity of the S-DAC in the presence of target ions, making it a suitable and selective fluorescent sensor for detecting Hg^2+^ and Fe^3+^ ions in aqueous media (Fig. 6(i) and (ii)).

#### Titration experiments

**Fig. 7:**
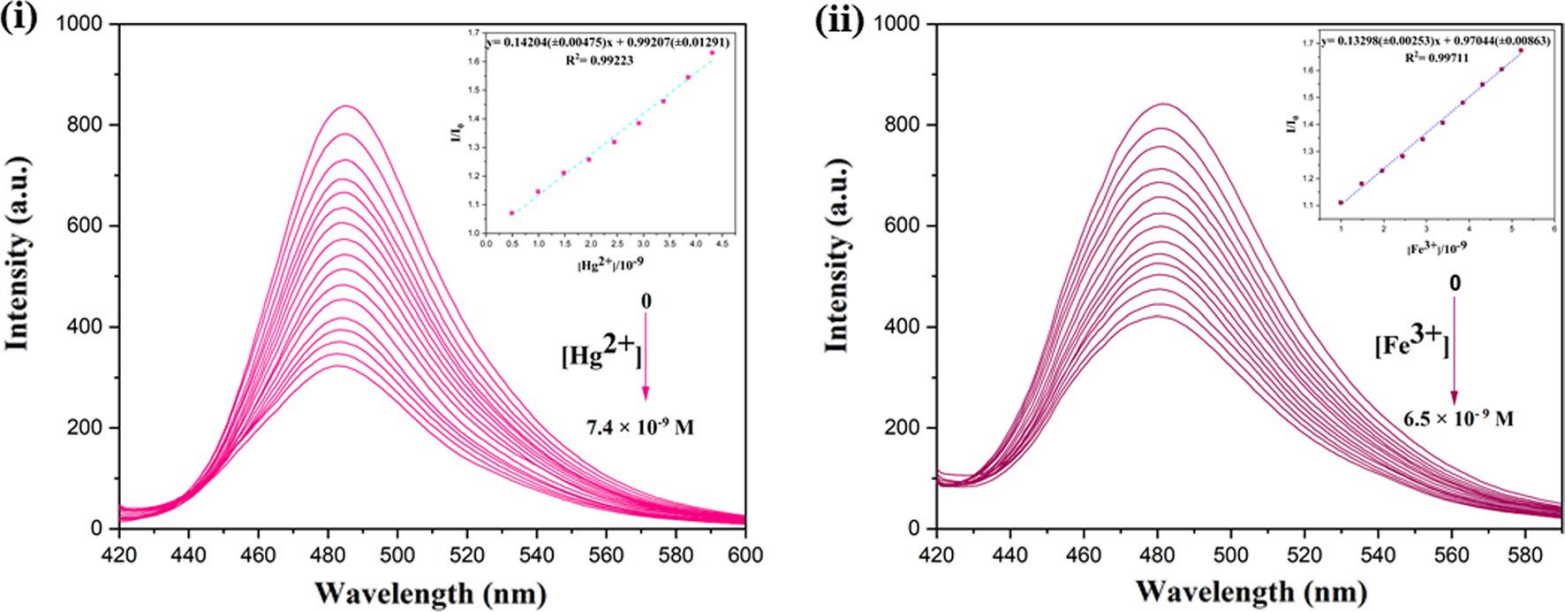
(**i**) Fluorescence emission of S-DAC after adding different concentrations of Hg^2+^ ion and (**ii**) Fluorescence emission of S-DAC after adding different concentrations of Fe^3+^ ion (The inset is the Stern–Volmer plot λ_ex_: 400 nm and λ_em_: 480 nm)

To evaluate the relation between fluorescence emission intensity and the concentration of Hg^2+^ and Fe^3+^ ions, titration studies were performed by different concentrations of Hg^2+^ and Fe^3+^ ions in the range of (0–200) × 10^–6^ M. The fluorescence emission intensity gradually declined upon adding target metal ions as demonstrated in Fig. 7(i) and (ii). Furthermore, the linear calibration curve was plotted for I_0_/I versus [Hg^2+^] and [Fe^3+^] (inset of (Fig. 7(i) and (ii)) and virtually indicated linearity between the emission intensity of S-DAC and the concentration of Hg^2+^ and Fe^3+^. The D_L_ = 3σ/m equation was also used to estimate the limit of detection (LOD) for S-DAC, where σ is the standard deviation intercept from the calibration curve, and m is the slope of the calibration curve. As a result, the detection limit was calculated as 0.28 × 10^–9^ mol L^−1^ for Hg^2+^ and 0.2 × 10^–9^ mol. L^−1^ for Fe^3+^ analytes. In addition, the linear concentration range was calculated as (0.49 × 10^–9^–4.39 × 10^–9^ mol L^−1^) and (0.99 × 10^–9^–5.21 × 10^–9^ mol L^−1^) for Hg^2+^ and Fe^3+^, respectively. The results of LOD reveal intense sensitivity of S-DAC toward Hg^2+^ and Fe^3+^ ions as an analyte. The Stern–Volmer Quenching constant was calculated as 0.14204 for Hg^2+^ and 0.13298 for Fe^3+^ analytes.

#### Quenching mechanism of S-DAC to Fe^3+^ and Hg^2+^ ions

**Scheme 2:**
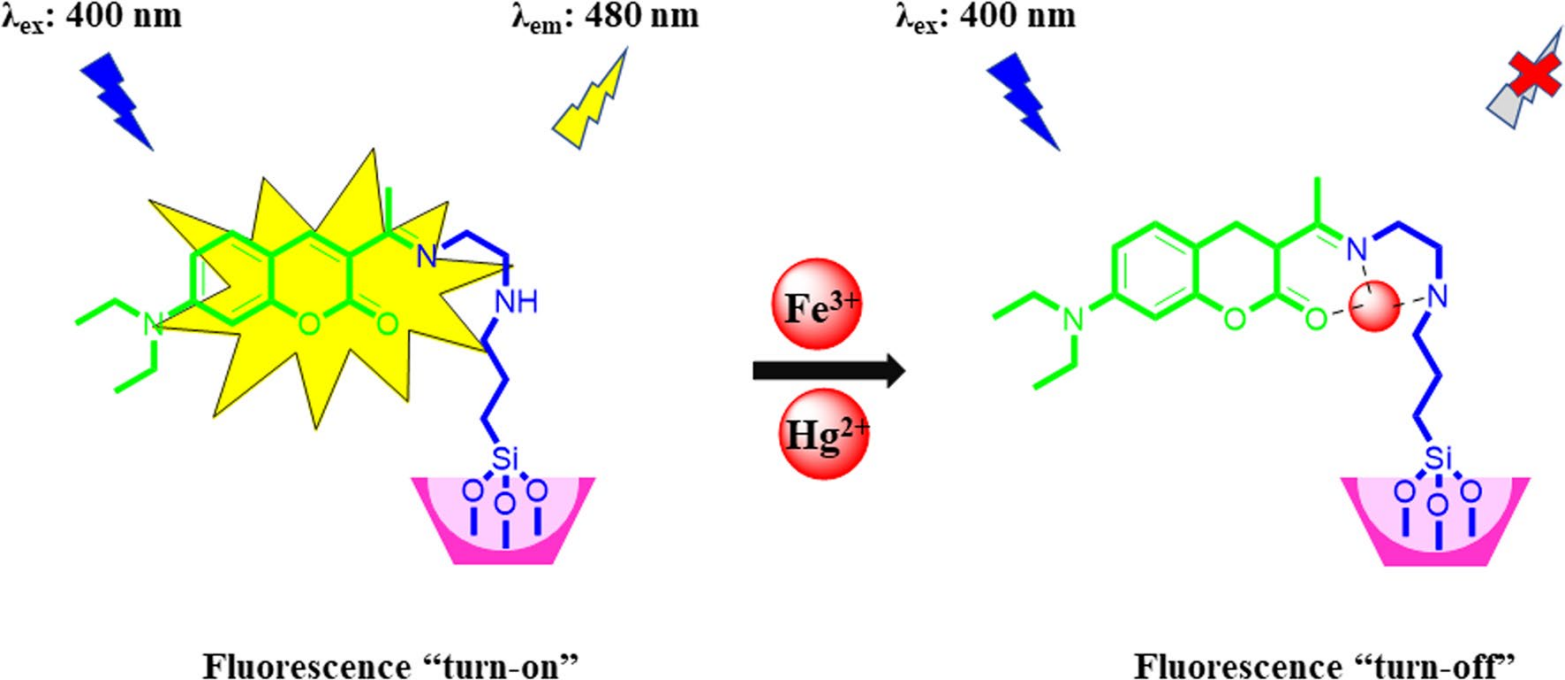
Proposed reaction of compound S-DAC with Fe^3+^ and Hg^2+^ ions

Fe^3+^ ions likely cause the fluorescence quenching of S-DAC due to their paramagnetic nature. The ferric ion has five unpaired electrons in the d orbitals, which significantly facilitates the transfer of electrons or energy, leading to the emission quenching of the fluorescent sensor. This transfer between Fe^3+^ and the fluorescent sensor prohibits the intersystem crossing, resulting in the non-fluorescence complex [58]. The fluorescence quenching upon adding the Hg^2+^ ion occurred probably due to the heavy atom effect [59]. The rotating orbiting electrons on the Hg^2+^ ion create a magnetic moment that can be coupled with the magnetic moment generated by the fluorophore electron spin, enabling the electrons to dissipate energy and relaxation to the ground state without emitting any light. The receptor Schiff-base in the S-DAC fluorescent sensor has three coordination sites: the ethylene diamine nitrogen atom, the carbonyl group's oxygen atom, and the imine group’s nitrogen atom. Hg^2+^ ions have a larger radius than other competitive cations, such as first-row transition metal cations, and also have a more substantial bond capability to the N atoms in the receptor than alkali, alkaline earth metal cations. These results in a stronger coordination bond of Hg^2+^ ions with the Schiff-base receptor. This process indicates that the S-DAC fluorescent sensor is a highly selective fluorescent sensor for Hg^2+^ in the water medium. The detailed quenching mechanism of the S-DAC fluorescent sensor in the presence of Hg^2+^ and Fe^3+^ ions is described in Scheme 2.

**Fig. 8:**
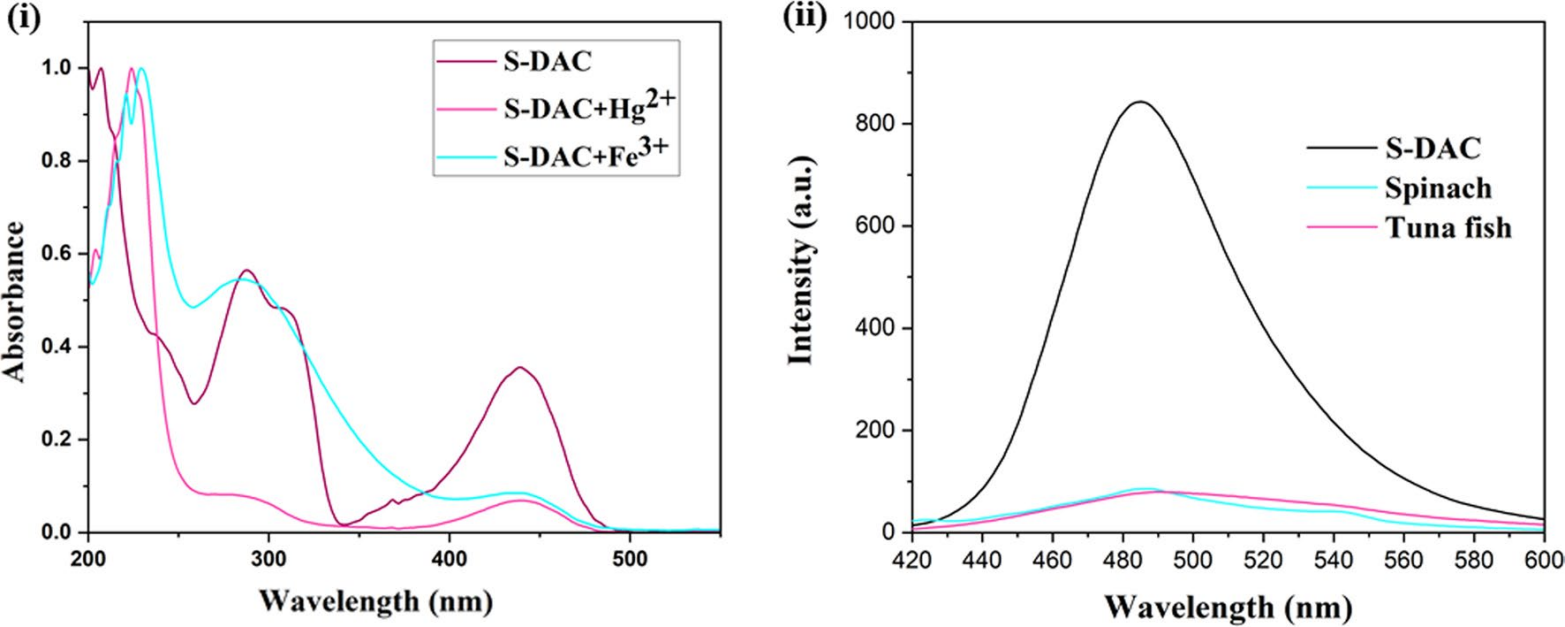
(**i**) The UV–Vis spectra of S-DAC in the presence and absence of iron and mercury ions and (**ii**) the fluorescence emission of S-DAC in the presence of spinach and tuna fish prepared solutions

To determine the mechanism, The UV–Vis spectra of S-DAC in the presence and absence of iron and mercury ions were investigated in the absence and presence of the analytes (Fig. 8i). The distinguished differences in the adsorption spectra of the samples confirm the static emission quenching mechanism, which is due to the formation of a complex between the fluorescent sensor and Fe^3+^ and Hg^2+^ ions [60]. To evaluate the feasibility of S-DAC as a fluorescent sensor in real samples, two samples of spinach and tuna fish were used to determine Fe^3+^ and Hg^2+^ ions, respectively. As explained in San et al. work [61], the digestion method was used to prepare the real samples. The fluorescence emissions of the fluorescent sensor were recorded in the absence and presence of the prepared samples. The measured fluorescence spectra of the S-DAC fluorescent chemosensor before and after adding the prepared samples was shown in Fig. 8ii. Therefore, S-DAC can be used as a fluorescent sensor to determine trace target ions in real samples, especially foodstuffs like spinach and tuna fish.

#### Logic gate and turn off–on behavior of S-DAC

**Fig. 9:**
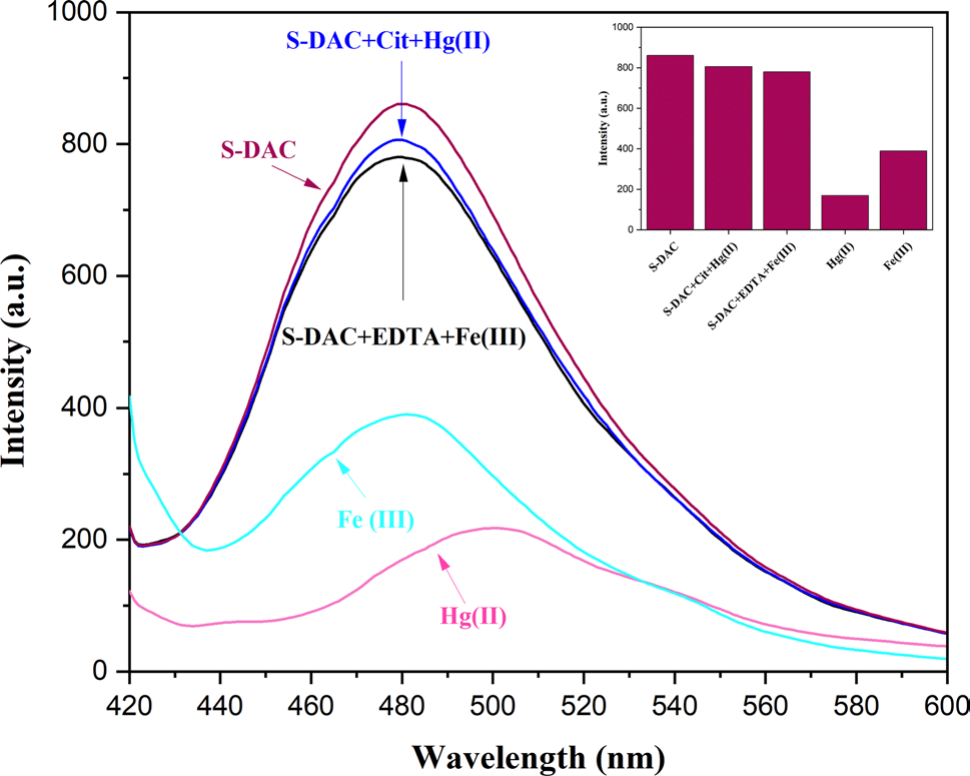
Turn off–on behavior of S-DAC in the presence of Hg^2+^, Fe^3+^, Trisodium citrate dihydrate, and EDTA

**Fig. 10:**
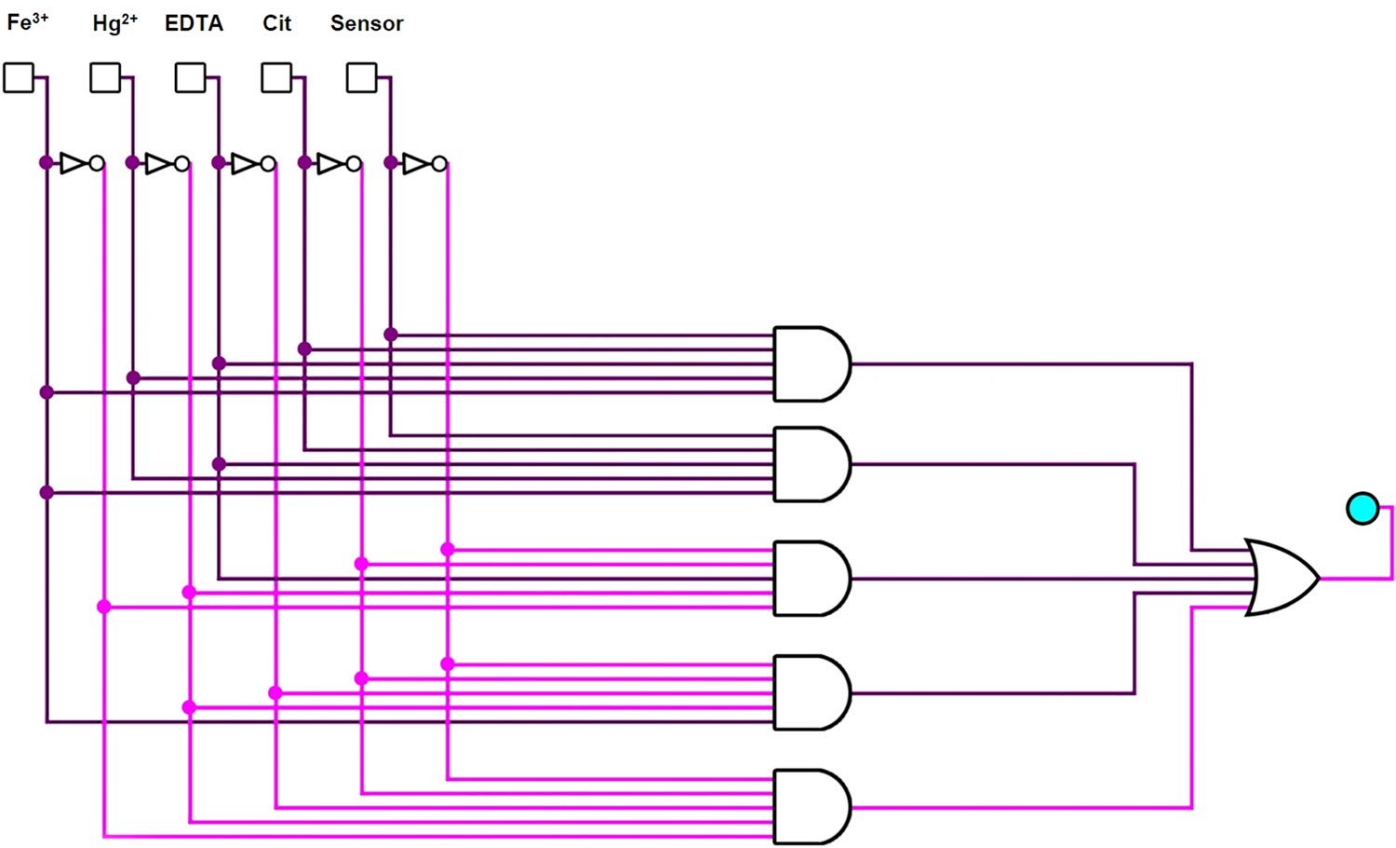
Logic circuit for S-DAC with S-DAC, Hg^2+^, Fe^3+^, Trisodium citrate dihydrate (Cit), and EDTA as the inputs

Further studies have revealed that adding trisodium citrate dihydrate (Cit) to the mixture of S-DAC and Hg^2+^ results in a significant enhancement in fluorescence emission intensity and the return to the initial emission state of the S-DAC. Without the presence of Hg^2+^ ions, no significant effect on the fluorescence emission of the S-DAC was observed. This phenomenon is likely due to the strong interaction between Hg^2+^ ions and trisodium citrate dihydrate, which can release the S-DAC fluorescent sensor by breaking the complex between the fluorescent sensor and Hg^2+^ ions, increasing the fluorescence emission intensity. The fluorescence emission intensity of the S-DAC–Fe^3+^ ions complex can be inverted by adding ethylenediaminetetraacetic acid (EDTA4Na). In contrast, the fluorescence emission of S-DAC has no response to (EDTA4Na) alone (Fig. 9). Similarly, as with Hg^2+^ and trisodium citrate dihydrate, the strong bonds between Fe^3+^ and EDTA could explain this observation. Accordingly, the logic circuit and truth table were designed with the trisodium citrate dihydrate, EDTA, Fe^3+^, and Hg^2+^ ions as input and fluorescence emission value as output, based on a standard expression called Sum-Of-Products. Regarding the output, ‘1’ and ‘0’ were called the emission quenching and fluorescence emission, respectively. The corresponding truth table and logic circuit diagram can be observed in Table 2 and (Fig. 10).

**Table 2:** The truth table for S-DAC

Hg (II)	Fe (III)	Cit	EDTA	S-DAC	Output (Quench)
0	0	0	0	1	0
1	0	0	0	1	1
0	1	0	0	1	1
0	0	1	0	1	0
0	0	0	1	1	0
1	1	0	0	1	1
0	1	1	0	1	0
0	0	1	1	1	0
0	1	0	1	1	1
1	0	1	0	1	1
1	0	0	1	1	0
1	1	1	0	1	1
1	1	0	1	1	1
1	0	1	1	1	0
0	1	1	1	1	0
1	1	1	1	1	0
0	0	0	0	0	0
1	0	0	0	0	0
0	1	0	0	0	0
0	0	1	0	0	0
0	0	0	1	0	0
1	1	0	0	0	0
0	1	1	0	0	0
0	0	1	1	0	0
0	1	0	1	0	0
1	0	1	0	0	0
1	0	0	1	0	0
1	1	1	0	0	0
1	1	0	1	0	0
1	0	1	1	0	0
0	1	1	1	0	0
1	1	1	1	0	0

#### Analysis

In addition, the performance of this chemosensor was compared to the others previously reported ones as listed in Table 3. It was demonstrated that this prepared chemosensor (S-DAC) releaved a higher sensitivity in detecting metal ions (Fe^3+^ and Hg^2+^) in aqueous media.

**Table 3:** Comparison between detection methods for Fe^3+^ and Hg^2+^ and this work

Sensor/probe	Detection method	Cations	Logic gate	LOD	Ref
Dihydropyrano quinoline	Fluorescent sensor	Fe^3+^	✗	2.0 µM	[46]
A Schiff base‐grafted	Fluorescent sensor	Hg^2+^	✗	7.34 nM	[62]
Diethyl 2-(9-Fluorenyl) Malonate Functionlized SBA-15	Fluorescent sensor	Fe^3+^	✗	1.99 µM	[63]
Schiff base immobilized on SBA-15	Fluorescent sensor	Fe^3+^	✗	1.49 mM	[64]
This work	Fluorescent sensor	Hg^2+^, Fe^3+^	✓	0.28 nM, 0.2 nM	

## Conclusion

A fluorescent sensor based on a coumarin derivative-functionalized nanoporous silica with high selectivity and sensitivity can be used for the detection of Fe^3+^ and Hg^2+^ ions in aqueous media with detection limits of 0.28 × 10^–9^ M and 0.2 × 10^–9^ M for Hg^2+^ and Fe^3+^, respectively. The fluorescence detection of target ions has occurred in the presence of other cations without any interference. The calibration curve indicates linearity between the emission intensity of S-DAC and the concentration of Hg^2+^ and Fe^3+^. As the concentration of the target ion increases and the complex forms between the sensor and the target ion, there is a gradual decrease in fluorescence emission. The quenching in fluorescence intensity with Hg^2+^ and Fe^3+^ ions can be returned to the original emission state, and enhancement in fluorescence emission of the S-DAC with trisodium citrate dihydrate and EDTA as masking agents, respectively. Accordingly, a sum-of-product logic gate was designed with the trisodium citrate dihydrate, EDTA, Hg^2+^, and Fe^3+^ as input and quenched fluorescence emission as the output. In addition, this sensor is used to detect Hg^2+^ and Fe^3+^ ions in real samples such as spinach and tuna fish.

### Supplementary Information

Supplementary Information (DOCX 2549 KB)

## Data Availability

The data supporting this study's findings are available from the corresponding author upon reasonable request.
