# Supplementary material for: Coumarin derivative-functionalized nanoporous silica as an on–off fluorescent sensor for detecting Fe3+ and Hg2+ ions: a circuit logic gate
Source: Discov Nano. 2024 Apr 22;19(1):70. doi: 10.1186/s11671-024-04013-9 (PMC11035537; doi:10.1186/s11671-024-04013-9)
Supplement: Supplementary file 1 — Supplementary Information (DOCX 2549 KB) [file 11671_2024_4013_MOESM1_ESM.docx]

**Coumarin derivative-functionalized nanoporous silica as an on-off fluorescent sensor for detecting Fe^3+^ and Hg^2+^ ions: a circuit logic gate.**

Zahra Mousavi ^a^, Jahan B. Ghasemi ^a^, Ghodsi Mohammadi Ziarani ^b^, Shahnaz Rahimi ^a^, Alireza Badiei ^a^, ***^*^***

^a^ School of Chemistry, College of Science, University of Tehran, P.O. Box: 14155-6455, Tehran, Iran

^b^ Department of Organic Chemistry, Faculty of Chemistry, Alzahra University, P.O. Box: 1993893973, Tehran, Iran

***^*^****E-mail address:* [abadiei@ut.ac.ir](mailto:abadiei@ut.ac.ir)

**Materials and reagents**

Tetraethylorthosilicate (TEOS), N-(2-Aminoethyl)-3-Aminopropyltrimethoxysilane, Pluronic P123, Piperidine, 4-Diethylamino salicylaldehyde, Ethyl acetoacetate, Ethyl acetate, Trisodium citrate dihydrate, Ethylenediaminetetraacetic acid (EDTA), EtOH, Toluene, metal nitrate salts, Sodium hydroxide, and Hydrochloric acid were purchased from Merck and Sigma- Aldrich companies and used without further purification.

**Characterization and instruments**

Low-angle X-ray scattering measurements were conducted using an X'Pert Pro MPD diffractometer with CuKα radiation (λ = 1.5418 Ǻ). Fourier transform infrared (FT-IR) spectra were obtained using a RAYLEIGH WQF-510 A. Thermogravimetric analysis (TGA) was performed using the STA503 model (BӒHR Thermoanalyse) in the temperature range of 25 to 1000 °C with a heating rate of 10 °C/min in air. N_2_ adsorption–desorption isotherm was recorded on a Micromeritics TriStar II plus at 77 K (liquid nitrogen temperature). All samples were degassed at 100 ˚C before the measurements. The Barrett–Joyner–Halenda (BJH) and Brunauer–Emmet–Teller (BET) equations were used to evaluate the physical properties of the samples, such as the specific surface area, pore volume, pore diameter, and pore size distribution. Photoluminescence (PL) spectra of the samples were obtained using an Agilent-G980A instrument. The UV-Vis spectra were collected using a Photonix Ar 2015. ^1^H and spectra were obtained using a VARIAN - INOVA 500 MHz model. Transmission electron microscopy (TEM) was carried out using PHILIPS, EM208S NETHERLAND with an accelerating voltage of 100 kV. The laser particle size analyzer and Zeta potential were obtained using a HORIBA - SZ100.

**General procedure for the synthesis of SBA-15**

The SBA‐15 mesoporous silica was synthesized using a previously reported procedure [1].

**General procedure for the synthesis of SBA-15 modified with N-(2-Aminoethyl)-3-Aminopropyltrimethoxysilane**

The SBA-Pr-NH-Et-NH_2_ compound was prepared according to the method mentioned in the reference [2]. First, SBA-15 (1 g) was dispersed in dry toluene (50 ml). Then, under stirring **N-(2-Aminoethyl)-3-Aminopropyltrimethoxysilane** (5 mmol) was added to the solution and refluxed for 24 hours. The obtained product was collected with a Buchner funnel, washed with toluene and ethanol, and finally dried at room temperature and coded as (SBA-Pr-NH-Et-NH_2_).

**General procedure for the synthesis of 7-diethylamino 3-acetyl coumarin (DAC)**

The DAC compound was prepared according to the method in reference [3], Scheme 1. The 4-diethylamino salicylic aldehyde (10 mmol) was dissolved in ethanol (20 ml). Then, under stirring, an equal molar amount of acetyl acetoacetate and piperidine (10 mmol) was added dropwise into the solution. The mixture was stirred at room temperature for 24 h. The final yellow crystalline precipitate was filtered, washed with ethanol, dried at room temperature, and coded as (DAC). ^1^H NMR (500 MHz, DMSO) δ 8.48 (s, 1H), 7.66 (d, *J* = 9.0 Hz, 1H), 6.79 (d, *J* = 9.0 Hz, 1H), 6.57 (s, 1H), 3.49 (q, *J* = 7.0 Hz, 4H), 2.51 (s, 3H), 1.14 (t, *J* = 7.0 Hz, 6H) (Fig. S1).





**Scheme. S1** The synthetic procedure of 7-diethylamino 3-acetyl coumarin (DAC)


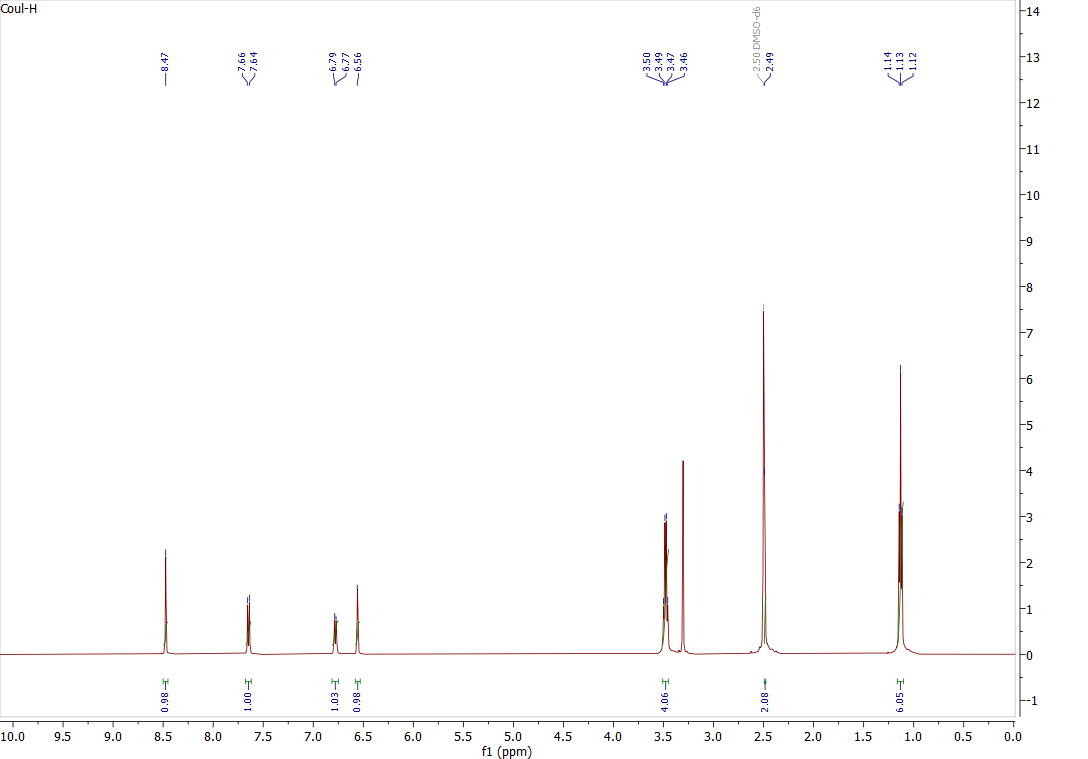


**Fig. S1** ^1^H NMR (500 MHz, DMSO) spectrum of compound DAC

**Effect of the pH**

The effect of pH as an essential variable in the performance of the fluorescent sensor should be studied. The fluorescence emission intensity changes of S-DAC in the absence and presence of Fe^3+^ and Hg^2+^ ions were carried out in the 2–10. The obtained results in Fig. S2 showed that below pH = 4, no fluorescence quench was observed. Indeed, in highly acidic pH conditions, the nitrogen of amino groups of S-DAC was protonated, which prevented the complexation reaction between the chelating agents and cited ions. Besides, in pH higher than 9, Fe^3+^ and Hg^2+^ hydroxide precipitation formed a dominant form, and the free Fe^3+^ and Hg^2+^ declined. As a result, the emission intensity was slightly quenched. Therefore, the best pH range for Fe^3+^ and Hg^2+^ detection was 4-9. However, S-DAC is an appropriate fluorescent sensor for detecting Fe^3+^ and Hg^2+^ in much higher alkaline waters, though relatively less efficient.

**
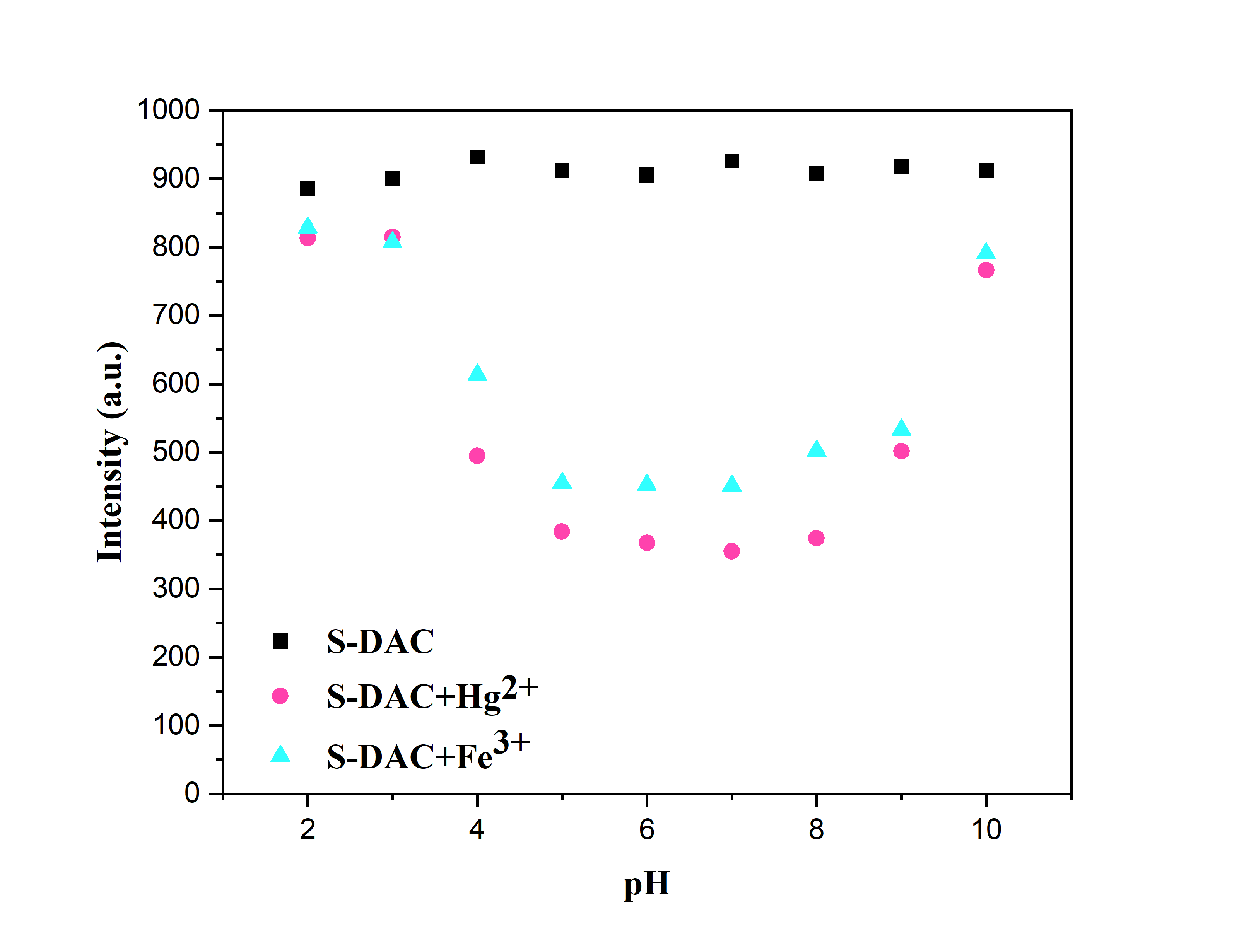
**

**Fig. S2** Effect of the pH on the fluorescence intensity of S-DAC suspension in the absence and presence of Fe^3+^ and Hg^2+^ solutions (λex = 400 nm, λem = 480 nm)

**References**

1. Bahrami, Z., A. Badiei, and G.M. Ziarani, *Thiol-functionalized mesoporous silica as nanocarriers for anticancer drug delivery.* Int. J. Bio-Inorg. Hybr. Nanomater, 2015. **4**(3): p. 121-128.

2. Zhang, Y., et al., *Synthesis of pyridyl Schiff base functionalized SBA-15 mesoporous silica for the removal of Cu (II) and Pb (II) from aqueous solution.* Journal of Sol-Gel Science and Technology, 2020. **94**: p. 658-670.

3. Bhattacharyya, A., S.C. Makhal, and N. Guchhait, *Evaluating the merit of a diethylamino coumarinderived thiosemicarbazone as an intramolecular charge transfer probe: efficient Zn (II) mediated emission swing from green to yellow.* Photochemical & Photobiological Sciences, 2019. **18**: p. 2031-2041.
